# Aerobic adaptation and metabolic dynamics of *Propionibacterium freudenreichii* DSM 20271: insights from comparative transcriptomics and surfaceome analysis

**DOI:** 10.1128/msystems.00615-24

**Published:** 2024-09-30

**Authors:** Iida Loivamaa, Annika Sillanpää, Paulina Deptula, Bhawani Chamlagain, Minnamari Edelmann, Petri Auvinen, Tuula A. Nyman, Kirsi Savijoki, Vieno Piironen, Pekka Varmanen

**Affiliations:** 1 Department of Food and Nutrition, University of Helsinki, Helsinki, Finland; 2 Department of Food Sciences, University of Copenhagen, Frederiksberg, Denmark; 3 Institute of Biotechnology, DNA Sequencing and Genomics Laboratory, University of Helsinki, Helsinki, Finland; 4 Department of Immunology, University of Oslo and Oslo University Hospital, Oslo, Norway; 5 Division of Pharmaceutical Chemistry and Technology, University of Helsinki, Helsinki, Finland; Istanbul Medipol University School of Medicine, Istanbul, Turkey

**Keywords:** *Propionibacterium*, vitamin B12, cobamides, aerobic, anaerobic, transcriptomics, surfaceomics

## Abstract

*Propionibacterium freudenreichii* (*PFR*) DSM 20271^T^ is a bacterium known for its ability to thrive in diverse environments and to produce vitamin B12. Despite its anaerobic preference, recent studies have elucidated its ability to prosper in the presence of oxygen, prompting a deeper exploration of its physiology under aerobic conditions. Here, we investigated the response of DSM 20271^T^ to aerobic growth by employing comparative transcriptomic and surfaceome analyses alongside metabolite profiling. Cultivation under controlled partial pressure of oxygen (pO_2_) conditions revealed significant increases in biomass formation and altered metabolite production, notably of vitamin B12, pseudovitamin-B12, propionate, and acetate, under aerobic conditions. Transcriptomic analysis identified differential expression of genes involved in lactate metabolism, tricarboxylic acid cycle, and electron transport chain, suggesting metabolic adjustments to aerobic environments. Moreover, surfaceome analysis unveiled growth environment-dependent changes in surface protein abundance, with implications for adaptation to atmospheric conditions. Supplementation experiments with key compounds highlighted the potential for enhancing aerobic growth, emphasizing the importance of iron and α-ketoglutarate availability. Furthermore, in liquid culture, FeSO_4_ supplementation led to increased heme production and reduced vitamin B12 production, highlighting the impact of oxygen and iron availability on the metabolic pathways. These findings deepen our understanding of *PFR*’s physiological responses to oxygen availability and offer insights for optimizing its growth in industrial applications.

**IMPORTANCE:**

The study of the response of *Propionibacterium freudenreichii* to aerobic growth is crucial for understanding how this bacterium adapts to different environments and produces essential compounds like vitamin B12. By investigating its physiological changes under aerobic conditions, we can gain insights into its metabolic adjustments and potential for enhanced growth. These findings not only deepen our understanding of *P. freudenreichii'*s responses to oxygen availability but also offer valuable information for optimizing its growth in industrial applications. This research sheds light on the adaptive mechanisms of this bacterium, providing a foundation for further exploration and potential applications in various fields.

## INTRODUCTION

*Propionibacterium freudenreichii* (*PFR*) is a Gram-positive bacterium belonging to the Actinomycetota phylum. It is commonly found in dairy products, particularly Swiss-type cheeses, where it plays a crucial role in shaping the flavor compounds and texture characteristics of the cheese (1, 2). The bacterium metabolizes lactate to produce propionic acid and carbon dioxide, which leads to the formation of gas bubbles responsible for the characteristic eyes in Swiss-type cheeses (3). Besides its role in cheese production, *PFR* has garnered significant attention due to its potential health benefits, including its immunomodulatory properties and the production of vitamin B12 (hereafter B12) (2, 4, 5).

B12, also known as cobalamin, is an essential nutrient vital for various physiological processes in humans (6). Unlike many other organisms, humans cannot synthesize B12 and must obtain it from dietary sources or microbial production (7). Notably, the synthesis of cobalamin, as well as the human inactive pseudovitamin-B12 (hereafter pseudo-B12), by *PFR* exhibits intriguing dependencies on environmental conditions, particularly oxygen availability. While the first steps of cobalamin synthesis follow the anaerobic pathway where cobalt is inserted at an early stage (8), the biosynthesis of the lower ligand, 5,6-dimethylbenzimidazole (DMBI), requires oxygen. The absence of oxygen during biosynthesis leads to the production of pseudo-B12 with adenine as the lower ligand (9).

*PFR* DSM 20271, the type strain of the species, has garnered significant attention due to its ability to produce the active form of B12 in various plant food matrices and under different applications (10–12). Furthermore, the response of DSM 20271 to oxygen and its ability to grow under aerobic conditions have attracted scientific interest. Recent studies have revealed that certain strains of *PFR*, including DSM 20271, while traditionally considered anaerobes, exhibit tolerance to oxygen. They can grow to high cell densities under mildly aerated conditions, which allow the consumption of oxygen and thereby keep levels of dissolved oxygen below the detection limit (13). The adaptation of DSM 20271 to these microaerobic conditions was shown to involve complex metabolic adjustments, affecting various cellular processes, including energy metabolism, redox balance, and gene expression (13, 14). *PFR* under aerobic conditions, including detectable oxygen concentrations, remains to be studied.

The genome sequence of DSM 20271 (15) indicates that the strain possesses enzymatic systems, such as superoxide dismutase and heme-containing catalase, which likely play crucial roles in protecting the bacterium against oxidative stress caused by oxygen exposure. Furthermore, like other *PFR* strains (16, 17), DSM 20271 is equipped with the genes required for aerobic respiration and for the complete pathway for heme synthesis. Heme and cobalamin synthesis is interconnected as both pathways begin with the formation of the uroporphyrinogen III precursor (18). 5-aminolevulinic acid is a common precursor of heme and cobalamin that is produced via the Shemin pathway (C4 pathway) and the C5 pathway (18). The heme biosynthesis in *PFR* has not been widely studied yet, but current evidence suggests that in *PFR*, 5-aminolevulinic acid is synthesized from glutamate via the C5 pathway (19). Since heme iron is more easily absorbed and has higher bioavailability than other forms of iron (20), bioprocessing of food materials with microbes producing heme potentially increases nutritional value by enhancing iron availability. The red color and flavor of meat are due to heme present in the muscles of animals (21, 22). Therefore, heme has attracted much attention as a key ingredient that mimics meat color and flavor in artificial meat in the food industry. For plant-based meat products, heme has been introduced using genetically engineered yeast or bacteria (22, 23).

In this study, we cultivated the DSM 20271 strain in bioreactors under control of the partial pressure of oxygen (pO_2_), keeping it at approximately 20% by sparging with pure oxygen/air as well as under anaerobic conditions by nitrogen gas sparging. Employing a comparative transcriptomic approach alongside comparative surfaceome analyses and targeted metabolite profiling, we sought to elucidate the bacterium’s response to oxygen. The findings of this study contribute to a deeper understanding of the physiological responses of *PFR* to oxygen availability and offer valuable insights for optimizing its growth. Furthermore, the identification of key compounds and metabolites enhancing the aerobic growth and colony-forming ability of *PFR* under aerobic conditions can have significant implications for industrial applications.

## MATERIALS AND METHODS

### Culture conditions

Strain DSM 20271 was grown in yeast extract–lactate-based medium (YEL) and routinely cultured at 30°C starting by streaking from glycerol stock (15%, −80°C) onto a solid medium (supplemented with 1.5% agar) and growing for 4 days in anaerobic jars (Anaerocult, Merck). Liquid cultures were obtained by inoculation of three separated colonies into 10 mL of liquid medium in 15-mL Greiner tubes and propagation for 3 days at 30°C without shaking. Experimental cultures were routinely inoculated to 1% (vol/vol) and 1.5% (vol/vol) in test tubes and bioreactors, respectively. All experiments were performed with three biological replicates.

### Cell density and viable cell count

Cell densities were assessed by measuring the optical density (OD) at a wavelength of 600 nm (OD_600_) by aseptically removing small aliquots from the bioreactors. This measurement was routinely taken during the 72-h bioreactor cultivations. Microbial counts were determined using the plate counting technique. Dilution series were prepared in sterile saline (0.9% NaCl) or PBS, and appropriate dilutions were either spread (100 µL) or spotted (10 µL) onto YEL agar plates. After incubation for 4–7 days in anaerobic conditions using Anaerocult (Merck) at a temperature of 30°C, colonies were counted, and the CFU/mL (colony-forming units per milliliter) were determined. The statistical significance of differences between final cell densities (measured at 600 nm (OD_600_) and as viable cell counts on agar (CFU/mL)) was analyzed using SPSS software [IBM SPSS Statistics 2021, Version 28.0.0.0 (190)] with independent-samples *t*-tests.

### Fermentation in bioreactors and sampling

To investigate the effects of available oxygen on gene expression, growth kinetics, and metabolic activity, batch fermentations were conducted in bioreactors under both aerobic and anaerobic conditions.

Under aerobic conditions, the partial oxygen pressure (pO_2_) was maintained at 20% throughout the cultivation process. This control was critical to address the significant impact of increasing cell density and fluid viscosity on oxygen transfer efficiencies, a concern that can be mitigated by controlling the pO_2_ level in bioreactor experiments (24). During anaerobic growth, the pO_2_ level was verified to be at 0% to ensure the absence of oxygen in the experiment.

The fermentations were carried out in Sartorius A bioreactors with a growth volume of 750 mL. The temperature was maintained at 30°C, and the pH was controlled at 7.0 using NaOH (5 M) and HCl (1 M). Stirring was set at 200 rpm for anaerobic experiments. In anaerobic conditions, continuous nitrogen gas flow at 20 cc was utilized, while in aerobic conditions, the oxygen saturation level was maintained at 20% through air and oxygen gas flow, with stirring alternating between 200 and 300 rpm.

Samples of 12 mL were taken aseptically in three biological replicates at four time points (I–IV) for each atmospheric condition. For anaerobic fermentation, the sampling points were at 12 hours (I), 26 hours (II), 32 hours (III), and 46 hours (IV). In aerobic fermentation, samples were collected at 13 hours (I), 40 hours (II), 52 hours (III), and 66 hours (IV). Surfaceome analyses were performed on samples from the initial time point (I) in both aerobic and anaerobic fermentations. For gene expression and ddPCR analysis, samples from time points I and III were analyzed.

Cell harvesting was conducted by centrifugation at 3,220 x *g* for 10 minutes at +4°C. The supernatants were collected and stored at −80°C for acid and vitamin analyses. Harvested cells for vitamin analysis were washed with 1 M Tris-HCl buffer (pH 8.0) and stored at −20°C.

For RNA sequencing, the harvested cells were immediately resuspended in 1,500 µL of RNA later (Invitrogen) and incubated overnight at +4°C. After incubation, the cells were harvested by centrifugation at 12,000 G for 5 minutes at +4°C and stored at −80°C.

### RNA extraction and purification

Cells stored at −80°C were thawed and resuspended in 50 µL of Tris-EDTA buffer (TE buffer). The cell suspension was then transferred to lysing matrix tubes containing glass beads. Cell lysis was performed using the FastPrep-24 homogenizer, with three cycles of 30 seconds each at a speed of 6.5 M/S. Between cycles, the samples were kept on ice for 60 seconds to maintain the temperature. After the final ice incubation, the lysates were suspended in 150 µL of TE buffer and thoroughly vortexed. Subsequently, the lysates were centrifuged at 12,000 *g* for 5 minutes at +4°C to remove cell debris and glass beads. The supernatants were carefully collected and stored at −20°C.

Total RNA extraction was carried out using the RNeasy Mini Kit (Qiagen) according to the manufacturer’s instructions, including DNase digestion to remove any remaining DNA. The RNA was eluted with 40 µL of nuclease-free water.

Following RNA extraction, the samples underwent DNAase treatment and were further purified using an RNA clean-up method. The concentration of purified single-stranded RNA was measured using a NanoDrop spectrophotometer. The RNA samples were divided into aliquots and stored at −80°C. To deplete ribosomal RNA (rRNA), the Ribo-Zero Plus rRNA Depletion Kit (Illumina Inc., USA) was employed, following the protocol provided by the manufacturer.

### RNA sequencing and data analysis

The RNA-seq libraries were generated using the QIAseq Stranded Total RNA Lib kit from Qiagen, and sequencing was performed on the Illumina NextSeq 500 platform.

To assess the quality of the sequencing reads, FastQC v0.11.7 was employed. Adapter sequences and low-quality reads (using parameters -m 30 -q 25) were removed using cutadapt v1.9.1. Reads that mapped to rRNA sequences were eliminated using sortmerna v2.1, while the remaining reads were aligned to the *PFR* DSM 20271 genome assembly GCF_000940845.1_ASM94084v1 using the mem algorithm of BWA v0.7.11. The number of reads mapped to each gene was counted using Htseq-count v0.1.11. Differential expression analysis was performed using DESeq2 (25), comparing atmospheric conditions (anaerobic versus aerobic) within growth phases (logarithmic and stationary). Additional detailed information on the validation of the RNAseq data with ddPCR is provided in the supplemental material.

Genes were considered to be significantly differentially expressed if their adjusted *P*-value (padj) was ≤0.05. Predicted Gene Ontology (26) terms for the genes were obtained using PANNZER2 (27) with default parameters. The lists of differentially expressed genes from the various comparisons were then examined for GO term enrichment across the Biological Process, Molecular Function, and Cellular Component ontologies using the enrichGO function from the R package clusterProfiler (v. 4.0) (28, 29). The reference set used for the analysis included all the genes annotated with GO terms in the genome. At sampling point I and sampling point III, GO categories were compared, and those showing statistically significant differences (*P* < 0.05), as determined by Fisher’s exact test, were listed under different conditions. The upregulated and downregulated genes within each GO category were specified. The later cut-off threshold was set at a ∣fold change∣ ≥2.0 (∣log2Fold∣ ≥1).

### UHPLC analysis of vitamin B12 and pseudo-B12

Analysis for the B12 and pseudo-B12 was based on ultra-high performance liquid chromatography (UHPLC) method described by Chamlagain et al*.* (30). B12 and pseudo-B12 were extracted in their cyano forms from cellular pellets using established methods, as detailed by Chamlagain et al. (30). Specifically, 0.1–0.2 g of the cell pellet underwent extraction with a pH 4.5 buffer (comprising 8.3 mM sodium hydroxide and 20.7 mM acetic acid) in the presence of sodium cyanide, yielding a 25-mL extract. The analysis was performed with Waters UPLC system (Waters, Milford, MA, USA) with a C18 column (Waters Acquity HSS T3, 2.1 × 100 mm, 1.8 µm) at a flow rate of 0.32 mL/min, a UV detection by a photodiode array (PDA) detector at 361 nm, and an injection volume of 5–15 μL was used. B12 and pseudo-B12 in the cyano forms were identified based on retention time and absorption spectrum and quantified against cyanocobalamin standard. Furthermore, LC-MS/MS was performed for the structural confirmation (31).

### HPLC method to analyze acids and glucose

Glucose, acetic acid (AA), lactic acid (LA), propionic acid (PA), pyruvic acid, and succinic acid were determined using a high-performance liquid chromatography (HPLC) method described in Chamlagain et al*.* (32) with modifications. Samples were centrifuged at 12,000 x *g* for 10 minutes, and the resulting supernatants were collected. After syringe filtration (Pall, MI, USA; 0.2 µm), the samples were stored at −20°C. Analyses were conducted on the thawed samples. The analysis was performed on a Hi-Plex H column (Agilent, CA, USA; 300 × 6.5 mm), with a HiPlex H guard column (Agilent, CA, USA; 50 × 7.7 mm). The HPLC system was equipped with a Waters 515 pump, autosampler, ultraviolet (UV) detector (Waters 717), and refractive index detector (HP 1047A, HP, USA). The mobile phase was 10 mM H₂SO₄, and the flow rate was set at 0.5 mL/min for 35 minutes with the column temperature maintained at 40°C.

### Quantitative heme analysis

The inoculated YEL was incubated at 30°C under microaerobic and anaerobic conditions for 4 days. To create a microaerobic condition, the cultures (20 mL) were grown in 100-mL glass bottles under shaking conditions (200 rpm) and opened once every day in a sterile environment. The medium was supplemented with 5 mg/L CoCl2, and in the case of Fe-supplemented samples, FeSO_4_ was also added (1 mM). After incubation, the cultures were centrifuged (6,800 × *g*, 10 minutes at room temperature). B12 and heme were measured in cells, while the supernatants were analyzed for the organic acid contents.

Samples for heme measurements were prepared as follows. A portion of the cell pellet (0.1 g) was suspended in 0.1 mL of 0.1 M NaOH (31) in lysing matrix tubes containing glass beads, and the cells were lysed using the FastPrep-24 homogenizer (five cycles of 30 seconds each at a speed of 4.5 M/s). To maintain the temperature, the samples were kept on ice for 60 seconds between the cycles. Another 0.1 mL of NaOH was added to the cell lysates, homogenized, and centrifuged (12,000 × *g* for 10 minutes at room temperature). The supernatants were carefully collected and analyzed for the heme concentration.

Heme concentrations were measured using a heme assay kit (MAK316, Sigma-Aldrich, St. Louis, MO, USA) according to the manufacturer’s instructions. Absorbance was measured at 405 nm. The concentration of heme in the samples was calculated against the heme calibrant. The heme content is reported as µg heme/g cells.

### Colony forming ability using spot-plating assay

*PFR* DSM 4902 and DSM 20271 cells were grown at 30°C in YEL liquid medium for 3 days until OD_600_ reached approximately 1.2. Cells were pelleted by centrifugation, washed once with PBS, and resuspended in PBS to the same OD_600_, serially tenfold diluted, and spotted (5 µL of dilutions) on YEL agar plates supplemented with appropriate chemicals. Plates were incubated under anaerobic or aerobic conditions at 30°C for 4 days (anaerobic) or 6 days (aerobic).

### Surfaceome analyses

#### Preparing the cell samples for proteomics and enzymatic cell-surface shaving

The surfaceome samples were harvested from logarithmic (I) growth phases of both aerobic (13-hour) and anaerobic (12-hour) fermentations by centrifugation (4 minutes, 4°C, 3,320 × g). The cells were washed with 0.1 M sodium acetate (pH 5.0, Sigma-Aldrich) and resuspended into 50 mM TEAB (17% sucrose) (triethylammonium bicarbonate buffer, Sigma-Aldrich) after centrifugation (4 minutes, 4°C, 3,320 × *g*). Enzymatic surface shaving was carried out with 0.05 µg/µL of trypsin (Promega, Madison, WI, USA) for 30 minutes at 37°C. Afterward, the protein–cell suspensions were first time centrifuged (4 min, RT, 4,000 × g) to ensure cell-free supernatant and second time centrifuged (2 minutes, RT, 16,000 × g) through Spin-X filters to recover the peptides and the enzyme. Flow-troughs were left to incubate for 17 hours at 37°C before the tryptic digestions were terminated with the addition of trifluoroacetic acid (TFA, Sigma-Aldrich) at a final concentration of 0.6%. The peptide concentrations were measured spectrophotometrically with NanoDrop-1000 (Thermo Fisher Scientific, DE, USA). Samples were stored at −20°C.

#### LC-MS/MS identification

The tryptic peptides were concentrated and purified using ZipTips C_18_ (Merck Millipore) according to the manufacturer’s instructions. An equal amount of each peptide sample was loaded into an Easy-nLC 1000 Nano-LC system (Thermo Scientific, Waltham, MA, USA) coupled with a quadrupole Orbitrap mass spectrometer (Q ExactiveTM, ThermoElectron, Bremen, Germany) equipped with a nano-electrospray ion source (EASY- SprayTM, Thermo Scientific, Waltham, MA, USA), and analyzed as previously described (33). The acquired MS raw files, for protein identification and label-free quantification (LFQ), were searched against an in-house database of DSM 20271^T^ (CP010341) (15) with MaxQuant (ver. 1.6.1.0) under the following settings: carbamidomethyl (C) was specified as a fixed modification; oxidation of methionine was set as a variable modification; a peptide tolerance was set at 20 ppm in the first search, and the main search error of 4.5 ppm was applied. Additionally, trypsin without the proline restriction enzyme option was used, allowing two miscleavages; the false discovery rate (FDR) filtering was set to 1% for both peptide and protein identification, and the minimal number of unique and razor peptides was defined as 1.

#### Surfaceome bioinformatics and statistics

The identified proteins were submitted to SignalP 5.0 (34), SecretomeP 2.0 (35), and LipoP 1.0 (36, 37) and (38) analysis tools to determine the presence of possible classical and nonclassical signal peptide sequences. Subcellular location was predicted with PSORTb 3.0.3 (39). Additionally, the prediction of protein transmembrane helices was acquired by TMHMM Server v. 2.0 (40, 41); general protein function annotation, according to the COG, was accomplished with EggNOG 5.0.0 (42); and the isoelectric points (pIs) and molecular weights (MWs) were predicted by EMBOSS Pepsats (43).

The statistical comparisons of the MaxQuant-derived protein identifications were performed in Perseus version 1.6.15.0 (44), at which the known contaminant, reverse hits, and proteins only identified by site were excluded. The MS-raw intensities were filtered to contain minimally two-third valid values in at least one of the growth environments to obtain the total and unique identifications. Statistical comparison

s of the normalized log2-transformed LFQ levels were also carried out in Perseus. The data were filtered to include proteins with valid LFQ-values in at least 70% of biological replicates in at least one group. Before a Student’s *t*-test with a permutation-based false discovery rate of 0.05, the missing values were imputed from a normal distribution using parameters: width 0.3 and downshift 1.8. A complete linkage hierarchical clustering was performed on normalized (Z-score) values. The mass spectrometry proteomics data have been deposited to the ProteomeXchange Consortium via the PRIDE (45) partner repository with the data set identifier PXD051703.

## RESULTS AND DISCUSSION

**Fig 1:**
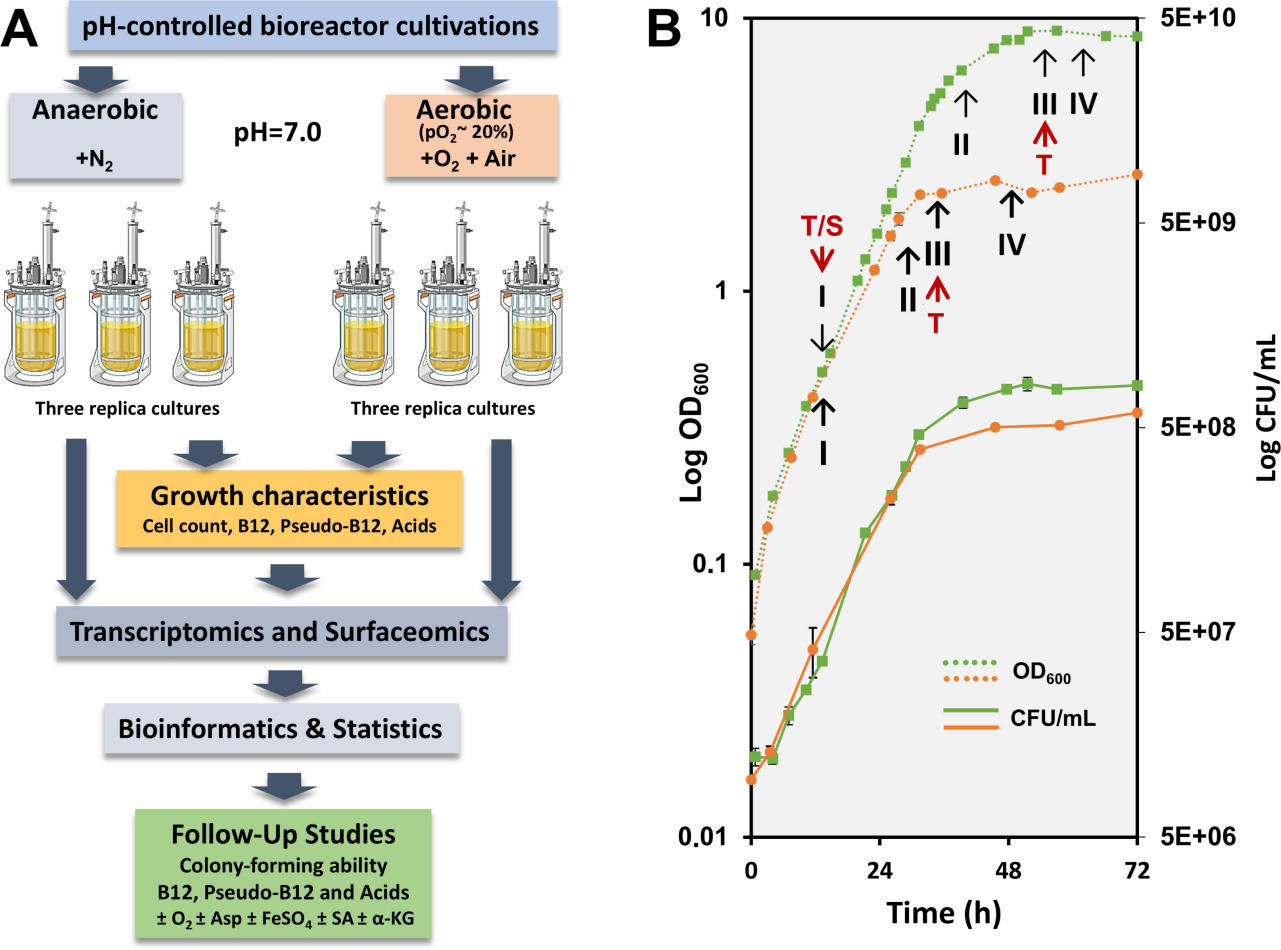
(**A**) A workflow illustrating the sequence of experiments, including bioreactor cultivations with studied parameters and follow-up growth experiments conducted in media supplemented with aspartate (Asp), succinate (SA), and α-ketoglutarate (α-KG). (**B**) Cell density (dotted line) and viable cell counts (solid line) in bioreactor-conducted experiments under aerobic (green) and anaerobic (orange) growth conditions. Sampling points for OD_600_ measurements and colony-forming unit determinations are marked with squares (aerobic) and circles (anaerobic), respectively. Key metabolite production dynamics and transcriptomic analyses were performed over four growth stages: mid-logarithmic (I), late logarithmic (II), early stationary (III), and late stationary (IV) phases. RNA sequencing was conducted on samples I and III. Red arrows indicate the specific time points/growth stages at which the cells were sampled for transcriptomic (T) and surfaceomic (S) analyses.

In the present study, we used pH-controlled bioreactor cultivations to investigate the cellular responses of DSM 20271 during growth under aerobic and anaerobic conditions. Our focus was on monitoring the central growth characteristics, the production of B12 and pseudo-B12 vitamins, and the major metabolic end products (Fig. 1A) at specific time points of growth and conditions (Fig. 1B). These findings were complemented with transcriptomic analyses and validated with label-free quantitative proteomics on cell surface proteins at the indicated time points (Fig. 1B). Additional follow-up studies were conducted to confirm the most relevant metabolic processes.

### Oxygen affects biomass, lactate metabolism, and formation of organic acids, B12, and pseudo-B12

#### Formation of biomass

To initiate the characterization of the response of DSM 20271 to aerobic growth, the strain was cultivated in bioreactors at a constant pO_2_ level of 20% and compared the growth characteristics to control cultivation under anaerobic control conditions (pO_2_ level below the detection limit). A pO_2_ level of 20% was selected based on preliminary experiments indicating that higher oxygen concentrations did not further increase cell density. A significant increase in biomass formation was observed under aerobic conditions with a pO_2_ level of 20% compared to anaerobic conditions. Specifically, the final cell densities, measured at 600 nm (OD_600_) and viable cell counts on agar (CFU/mL), were 3.2 and 1.4 times higher (*P* < 0.05), respectively, after 72 hours of cultivation under aerobic conditions (Fig. 1B).

#### Secretion of organic acids

**Fig 2:**
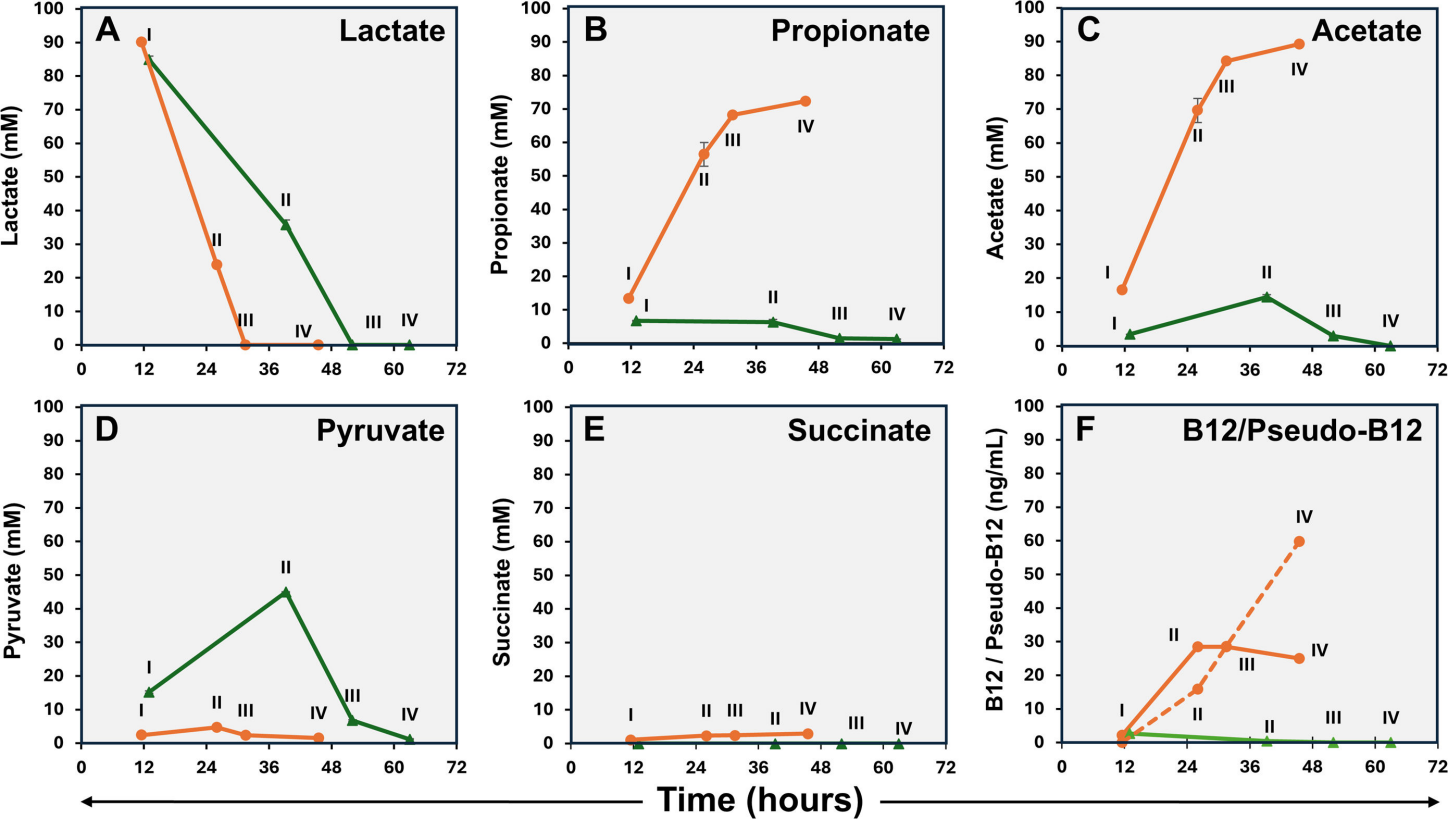
Lactate utilization (**A**), excreted propionate (**B**), acetate (**C**), pyruvate (**D**), succinate (**E**), and intracellular vitamin B12 and pseudo-B12 (F; vitamin B12 – solid line, pseudo-B12 – dashed line) of *PFR* DSM 20271 during cultivation under aerobic (green) and anaerobic (orange) fermentations performed in bioreactors. *N* = 3, except pseudo-B12 samples III and IV: *n* = 2. No pseudo-B12 was detected under aerobic growth conditions.

Lactate was found to be completely depleted from the medium after 32 hours and 52 hours under anaerobic and aerobic conditions, respectively, indicating faster lactate consumption under anaerobic conditions (Fig. 2A). Under anaerobic conditions, the levels of propionate and acetate in the growth medium increased over time, especially during logarithmic growth (Fig. 2B and C). In contrast, under aerobic conditions, only low levels of propionate were detected in logarithmic phase samples, with levels dropping below the limit of detection in stationary-phase samples. Acetate levels followed a similar pattern under aerobic conditions, being fully depleted from the medium after an initial increase (Fig. 2C). During logarithmic growth, the pyruvate levels in the media were higher under aerobic conditions but decreased to low levels in stationary-phase samples (Fig. 2D), whereas succinate was only detected in growth media in samples from anaerobic conditions (Fig. 2E). Pyruvate in culture supernatants, at concentrations reflecting its intracellular levels in *PFR*, has been reported previously (46), but to the best of our knowledge, there are no reports of the effect of atmospheric conditions. As the elevated growth under aerobic conditions is accompanied by diminished lactate consumption and, on the other hand, increased pyruvate excretion, it is tempting to assume that ATP production is maintained by upregulation of the later stages of the aerobic respiration pathway. The accumulation of succinate during adaptation to anaerobic or oxygen-limiting conditions has been previously observed in *Mycobacterium tuberculosis* (47).

#### Synthesis of B12 and pseudo-B12

B12 and pseudo-B12 levels were analyzed in cellular and supernatant (media) samples. However, under the conditions used, cobamides could only be detected from cell samples, and no B12 or pseudo-B12 was detected in the media of either aerobic or anaerobic cultivations. Under aerobic conditions, the concentration of B12 in the cells was very low at each sampling point and below the detection limit at sampling points II–IV (Fig. 2F). Under anaerobic conditions, B12 reached 28.5 ng/mL as fermentation progressed, while the concentration of pseudo-B12 increased as the fermentation process approached completion and reached 59.8 ng/mL. (Fig. 2F). It is noteworthy that cultivation of this strain in our bioreactors under identical conditions but with no gas sparging, thus resembling microaerobic conditions, resulted in superior B12 production. The levels were 1.7 to 4.9 times higher compared to those of anaerobic conditions, along with a distinct metabolite production pattern (Fig. S1).

In a previous study (13), the DSM 20271 strain was cultivated in bioreactors under conditions with an oxygen supply at a level that allowed its concentration to drop below the detection limit in the culture through consumption by cellular activity. Like those microaerobic conditions, aerobic conditions used here were found to increase biomass production compared to anaerobic conditions. However, the metabolite production profile under aerobic conditions seems to differ from those reported under microaerobic conditions. First, lower oxygen concentrations promoted propionate production during the lactate consumption phase, followed by its oxidation to acetate (13) (Fig. S1). Under the more aerobic conditions used here, the consumption of lactate from the media was not accompanied by propionate accumulation. Furthermore, the B12 production appears to be drastically affected by increasing oxygen concentration, as microaerobic conditions increased B12 production compared to anaerobic conditions (14) (Fig. S1), which is opposite to what was observed here under aerobic conditions (Fig. 2F). The ratio of B12 to pseudo-B12 in microaerobic conditions was not analyzed in previous studies due to the use of a microbiological assay that does not distinguish between them (14). In this study, we observed that the active form of B12 is produced under microaerobic conditions, whereas very low levels of either B12 or pseudo-B12 are produced under aerobic conditions (Fig. 1; Fig. S1). Thus, the typical features associated with *PFR* as an efficient producer of B12 and short-chain fatty acids, such as propionate and acetate, do not apply under aerobic growth conditions.

### Transcriptomics of DSM 20271 under anaerobic and aerobic conditions

In this study, we used gene expression data to identify genes critical for growth under both aerobic and anaerobic conditions, with a focus on genes contributing to the growth of *PFR* under aerobic conditions, as the physiology and the gene expression of this species in aerobic environments have not been extensively studied (13). We compared the gene expression of strain DSM 20271 grown under aerobic and anaerobic conditions by isolating total RNA from cells at two distinct growth phases: sampling point I, representing the logarithmic growth phase, and sampling point III, representing the stationary phase. In the following comparisons, upregulation and positive fold change (FC) values refer to higher expression in aerobic conditions compared to anaerobic conditions, while downregulation and negative FC values indicate higher expression in anaerobic conditions compared to aerobic conditions. These samples were then subjected to RNA sequencing to assess differences in gene expression across these growth stages.

**Fig 3:**
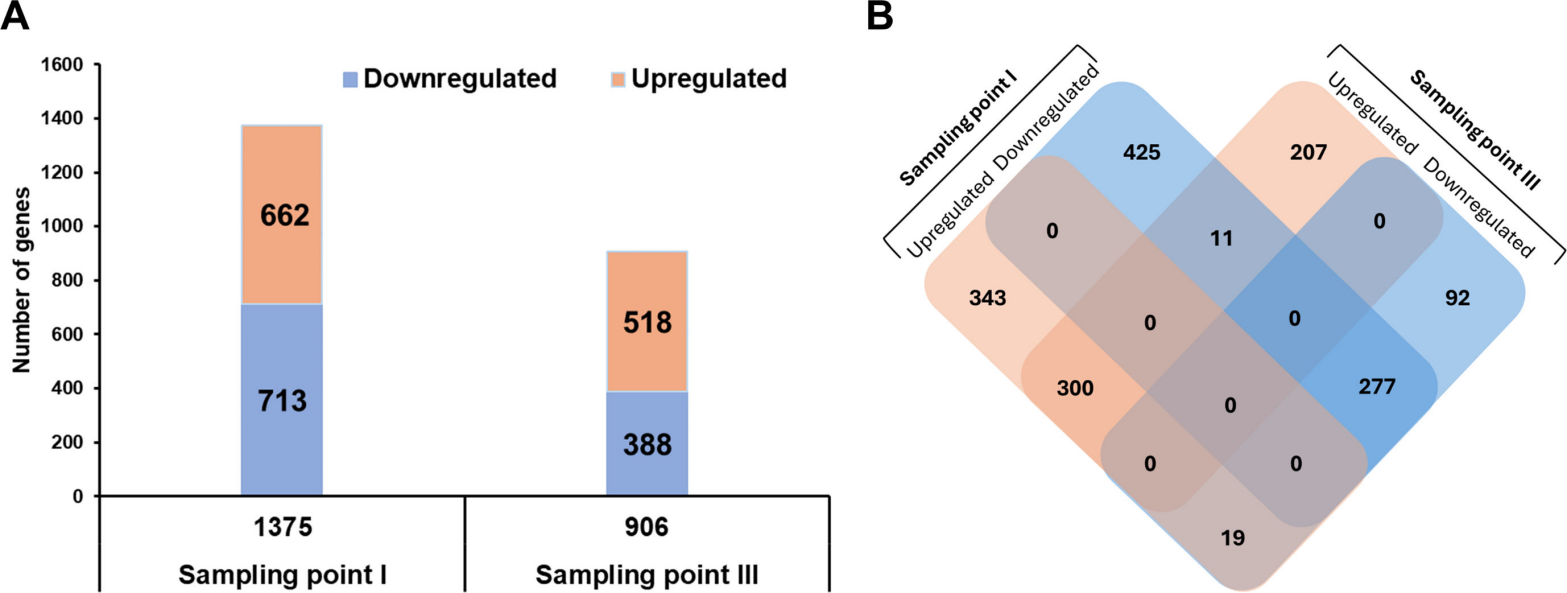
Differential expression of genes in *PFR* DSM 20271 across two sampling points under aerobic versus anaerobic growth conditions. (**A**) A total of 1,375 genes exhibited significant differential expression (adjusted *P*-value < 0.05), with 713 genes downregulated and 662 upregulated at sampling point I, and 906 genes were differentially expressed at sampling point III, including 388 downregulated and 518 upregulated. (**B**) Venn diagram illustrating the overlap of differentially expressed genes (DEGs) at the two sampling points. Sampling point I coincides with the logarithmic growth phase, while sampling point III corresponds to the stationary phase. Of the 662 upregulated DEGs at sampling point I, 300 remained upregulated and 19 were downregulated at sampling point III. Additionally, 277 of the 713 downregulated DEGs at sampling point I remained downregulated at sampling point III, and 11 were upregulated.

Pairwise differential expression analyses were performed to identify specific differences between conditions at each growth phase. A total of 1,375 genes (59.3% of total genes) were found to be differentially expressed at sampling point I, with an adjusted *P*-value (padj) < 0.05. Among these, 713 genes (51.9%) were downregulated, and 662 genes (48.1%) were upregulated under aerobic conditions. Additionally, 906 genes (39.1% of total genes) exhibited differential expressions at sampling point III. Of these, 388 genes (42.8%) were downregulated, and 518 (51.2%) genes were upregulated under aerobic conditions (Fig. 3A). A comprehensive list of differentially expressed genes, including annotation results and expression levels under anaerobic and aerobic conditions at sampling points I and III, is provided in Table S1. Of the 662 upregulated DEGs at sampling point I, 300 were also upregulated at sampling point III, while 19 were downregulated at sampling point III. Furthermore, 277 of the 713 downregulated DEGs at sampling point I remained downregulated at sampling point III, and 11 were upregulated (Fig. 3B). The specific lists of these upregulated and downregulated genes are provided in Table S2.

### Gene ontology (GO) enrichment analysis

A GO enrichment analysis was conducted to gain a comprehensive understanding of the functional groups represented by the differentially expressed genes (DEGs). This analysis compared the aerobic and anaerobic growth conditions at sampling points I and III. GO terms that were found to be overrepresented with statistical significance (*P* < 0.05) were analyzed in detail.

At sampling point I, 232 out of 1,375 DEGs were assigned to various GO terms, while at sampling point III, 216 out of 906 DEGs were categorized into GO terms. At sampling point I, the significant GO categories included transmembrane transport, transmembrane transporter activity, ATPase activity, zinc ion binding, magnesium ion binding, iron–sulfur cluster 4Fe-4S binding, “*de novo*” IMP biosynthetic process, transferase activity (transferring glycosyl groups), and carbohydrate metabolic process. Similarly, at sampling point III, the DEGs were classified into 11 significant GO categories (*P* < 0.05), including structural constituent of ribosome, translation, rRNA binding, ribosome, plasma membrane, tRNA binding, glycolytic process, DNA recombination, peptidoglycan biosynthetic process, cytoplasm, and transferase activity (transferring acyl groups).

The list of genes categorized into each GO group, along with the number of genes upregulated or downregulated under aerobic conditions, is provided in Table S3.

### Lactate metabolism

**Fig 4:**
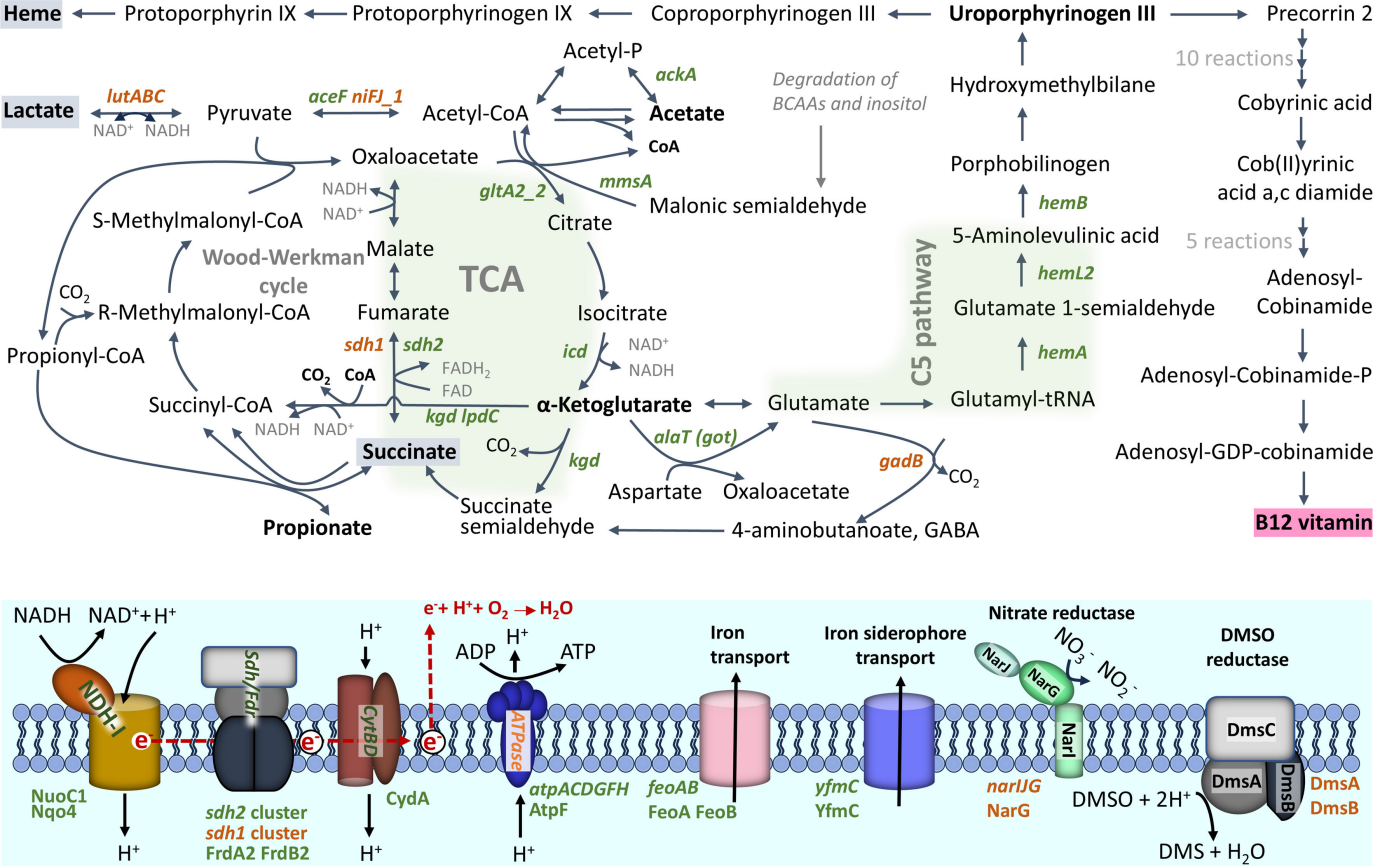
Schematic view of metabolism during aerobic and anaerobic growth. The presentation of the Wood–Werkman and TCA cycles was adapted from (50–52). Differentially expressed genes (≥ twofold difference, adjusted *P*-value < 0.05) are shown in green (increased aerobically) and orange (increased anaerobically). The pathways with the most differentially expressed genes, the TCA cycle and the C5 pathway for porphyrin synthesis, are highlighted with a green background. A tentative diagram of the aerobic electron transport chain, consisting of NADH dehydrogenase/complex I (NDH-I), succinate dehydrogenase/fumarate reductase (SDH/FDR), cytochrome bd complex (CytBD), and ATP synthase (ATPase), is shown, as well as the predicted transmembrane transport systems for iron and iron siderophore with detected with higher abundance under aerobic condition RNA-seq and surfaceome analyses. Putative membrane spanning structures of nitrate and DMSO reductases possibly involved in anaerobic respiration are shown. Differentially abundant proteins (FDR 0.05) detected in surfaceome analysis are indicated using green/orange fonts.

Analyses of organic acids from bioreactor cultivations indicated that lactate consumption was more rapid under anaerobic conditions compared to the aerobic atmosphere (Fig. 2A). The gene RM25_RS08745, encoding L-lactate permease, is the first gene within a predicted four-gene operon (*glcA-lutABC*, RM25_RS08730 – RM25_RS08745). This operon contains genes encoding widely conserved lactate utilization proteins (A, B, and C), mediating the oxidative conversion of L-lactate into pyruvate (Fig. 4) (48, 49). Here, the expression of this operon was upregulated under anaerobic conditions at both sampling points (I and III). Specifically, in the III samples, the expression fold changes range from 2.4 to 3.9, correlating with the observed lactate consumption. Regardless of atmospheric conditions, the lactate utilization operon was downregulated upon transition to the stationary phase.

Among other genes encoding proteins predicted to be involved in lactate utilization, RM25_RS01945, which encodes a putative FAD-dependent D-lactate dehydrogenase, was expressed 3.8-fold higher under anaerobic conditions compared to aerobic conditions at sampling point III. These findings suggest that lactate metabolism is more active under anaerobic conditions. Multiple pathways are available for the conversion of pyruvate into acetyl-CoA. Two enzymes, the pyruvate dehydrogenase (PDH) complex and pyruvate ferredoxin oxidoreductase (PFOR), can perform this conversion. In this study, one of the two paralogs encoding pyruvate ferredoxin oxidoreductase, *nifJ1* (RM25_RS00920), displayed higher expression (2.8-fold) under anaerobic conditions in sample point III, whereas only one predicted component of pyruvate dehydrogenase complex showed significantly differential expression, namely, RM25_RS07455 (*aceF*), with twofold higher expression under aerobic conditions in sample point III (Table S1; Fig. 4). The accumulation of pyruvate in the medium during aerobic and its absence under anaerobic conditions (Fig. 2D) indicate that pyruvate is metabolized more efficiently under anaerobic conditions, with the expression data suggesting that this could be due to increased PFOR activity.

Notably, the *bkdA-bkdB-phdC* gene cluster (RM25_RS01015, RM25_RS01020, RM25_RS01025), encoding the branched-chain α-ketoacid dehydrogenase complex (BCKDC), showed significantly higher expression levels (2.0–8.6-fold) under aerobic conditions at both sampling points (I and III). BCKDC is involved in the catabolism of branched-chain amino acids (BCAA), which can produce malonic semialdehyde, a substrate of the acetyl-CoA-producing enzyme methylmalonate–semialdehyde dehydrogenase (MmsA). The *mmsA* gene (RM25_RS08965) was found to be expressed at a 5.3-fold higher level under aerobic conditions in sample point I compared to anaerobic conditions (Table S1; Fig. 4).

### Propionate metabolism, TCA cycle, and respiratory complexes

The gene cluster RM25_RS08835 to RM25_RS08850 encodes the key enzyme, a multimeric transcarboxylase (EC 2.1.3.1), methylmalonyl-CoA carboxytransferase (MMC), of the propionate-forming Wood–Werkman cycle (53). Interestingly, this gene cluster was found to be constitutively expressed under the conditions investigated in this study (Table S1). This finding is consistent with that of a previous study by Brzuszkiewicz et al. (54), which also observed constitutive expression of the MMC genes at both logarithmic and stationary growth phases in the closely related bacterium *Cutibacterium acnes*. Additionally, it aligns with the study conducted by Dank et al. (13), where proteins involved in the Wood–Werkman cycle were found to be as abundant in propionate consumption as in lactate consumption phases in *PFR*.

The TCA cycle utilizes acetyl-CoA and converts NAD+ to NADH as part of its metabolic reactions. The genes encoding allosterically regulated TCA enzymes at critical control points, such as citrate synthase (*gltA2_2*, RM25_RS11475), isocitrate dehydrogenase (*icd*, RM25_RS03180), as well as α-ketoglutarate decarboxylase (*kgd*, RM25_RS05675) and dihydrolipoyl dehydrogenase (*lpdC*, RM25_RS05360) of α-ketoglutarate dehydrogenase complex, exhibited higher expression levels under aerobic conditions in at least one of the sampling points (I and III) (Table S1; Fig. 4). While previous research has demonstrated the simultaneous activity of the Wood–Werkman and TCA cycles (50), the present results indicate that aerobic conditions promote a shift in the central carbon metabolic pathway toward TCA cycle activity, contributing to the utilization of acetyl-CoA and the generation of NADH (Fig. 4).

The fermentation of pyruvate by propionibacteria typically produces propionic and acetic acids, as well as significant amounts of other products such as succinate (55). The primary function of the succinate dehydrogenase (SDH) complex in bacteria is to catalyze the oxidation of succinate to fumarate while transferring electrons to the electron transport chain. The genome of DSM20271 harbors two gene clusters encoding the subunits of the SDH complexes. These clusters consist of three genes each: RM25_RS06150, RM25_RS06155, and RM25_RS06160 and RM25_RS06655, RM25_RS06660, and RM25_RS06665, named *sdh1* and *sdh2*, respectively.

The *sdh* gene clusters exhibited distinct patterns of gene expression under different atmospheric conditions. Specifically, the *sdh1* cluster demonstrated a significantly higher expression level (2.8–3.4-fold increase) during anaerobic conditions (I and III samples). Conversely, at sampling point III, the RM25_RS06655 and RM25_RS06660 genes of the *sdh2* cluster exhibited significantly higher expression levels (2.3- and 2.8-fold increase) under aerobic conditions (Table S1; Fig. 4). Based on the different expression patterns observed, we propose that the *sdh2* cluster is responsible for encoding SDH, catalyzing the conversion of succinate to fumarate, and is involved in both the TCA cycle and aerobic respiration. In contrast, the *sdh*1 cluster encodes fumarate reductase, which reduces fumarate to succinate during anaerobic respiration. This is consistent with the accumulation of succinate in the medium under anaerobic conditions and its absence from the medium under aerobic conditions. These two sets of SDH/fumarate reductase complexes have been reported earlier in various studies on *PFR* (13, 16, 51), providing further support to our findings. Additionally, it has been suggested, but not yet explored, that in *Acidipropionibacterium acidipropionici,* the other SDH complex could function in anaerobic fermentation as fumarate reductase and another complex in aerobic fermentation as SDH (51).

The expression of the NADH dehydrogenase complex, encoded by the gene cluster RM25_RS02335 to RM25_RS02400, and the expression of the cytochrome-bd oxidase, encoded by genes RM25_RS00880 to RM25_RS12185, were not significantly influenced by atmospheric conditions. However, the *atpACDGFH* genes of cluster RM25_RS05560 to RM25_RS05595, which encodes the ATP synthase enzyme complex, demonstrated higher expression levels under aerobic conditions, particularly in the later sampling point (III). This indicates that ATP synthesis is more efficient in the presence of oxygen. These findings align with those of the conducted study by Dank et al. (13), where they observed that the respiratory pathway remains consistently active under both anaerobic and microaerobic conditions.

The expressions of the NADH dehydrogenase complex, the cytochrome-bd oxidase, and the ATP synthase complex were found to be differentially affected by the growth phase. The gene cluster of the NADH dehydrogenase (RM25_RS02335 to RM25_RS02400) and the ATP synthase enzyme (RM25_RS05560 to RM25_RS05595) complexes showed downregulation at the onset of the stationary phase. In contrast, the cluster of cytochrome-bd oxidase (RM25_RS00880 to RM25_RS12185) was either constitutively expressed under both atmospheric conditions and, in the case of RM25_RS00880, exhibited a 2.5-fold increase in expression at the stationary phase under anaerobic conditions. Genes of cluster RM25_RS10135 to RM25_RS10150 encoding transmembrane respiratory nitrate reductase exhibited clear upregulation, up to 17-fold, under anaerobic conditions at sampling point I, indicating anaerobic respiration activity with nitrate as the electron acceptor (Table S1; Fig. 4).

### Synthesis of B12 and heme

B12 and heme are tetrapyrrols that play crucial roles in various biological processes. B12 serves as an essential coenzyme for enzymatic reactions, particularly in the metabolic pathways involved in amino acid and nucleotide synthesis (56). Heme, however, is involved in important cellular oxidative metabolic pathways, such as oxygen transportation, response to oxidative stress, and oxidative phosphorylation. Here, we observed a significant effect of atmospheric conditions on cobalamin production by DSM 20271 in bioreactor cultivations. The cobalamin biosynthesis genes in *PFR* are mainly located in three clusters: RM25_RS02055 to RM25_RS02075, RM25_RS03590 to RM25_RS03625, and RM25_RS04745 to RM25_RS04770. Cluster RM25_RS02055 to RM25_RS02075 forms the locus for cobalt transport, while RM25_RS03590 to RM25_RS03625 and RM25_RS04745 to RM25_RS04770 encode the assembly and activation of the corrin ring, respectively (16, 57). Additionally, there are single genes, such as RM25_RS02940 (*bluB/cobT2*), RM25_RS03660 (*cobQ2*), and RM25_RS05545 (*cobA*), involved in cobalamin biosynthesis. Under aerobic conditions during the later sampling point (III), the genes RM25_RS02070 (*cbiN*) and RM25_RS02075 (*cbiM*) encoding cobalt transport proteins were significantly upregulated (4.7- and 3.2-fold, respectively), while the expression of all the other B12 biosynthetic genes was not significantly affected by the atmospheric conditions. The regulatory mechanism of the B12 biosynthetic pathway is known to involve cobalamin riboswitches, predicted to be located upstream of RM25_RS03590 (*cbiL*), RM25_RS04770 (*cobD*), and RM25_RS02075 (*cbiM*) (58, 59). Cobalamin riboswitch controls gene expression through transcriptional and/or translational modifications (60, 61). Notably, the function of riboswitch upstream of *cbiM* was recently characterized in *PFR* strain P.UF1, which revealed control at the transcriptional level (58). Our results support this, indicating significantly differential transcription of the *cbiM* and *cbiN* genes under the conditions used. On the other hand, the differential production of B12 and the constitutive expression of other B12 genes under the studied conditions suggest regulation by mechanisms other than transcriptional control.

5-Aminolevulinic acid (ALA) is a precursor metabolite involved in the biosynthesis of tetrapyrrole compounds, which in *PFR* is synthesized via the C5 pathway (19, 62, 63). In this pathway, ALA is synthesized from glutamate through the coordinated actions of glutamyl-tRNA synthetase (encoded by *gltX*), glutamyl-tRNA reductase (encoded by *hemA*), and glutamate semialdehyde aminotransferase (HemL). HemB converts two ALA molecules into porphobilinogen (PBG), which is further polymerized into hydroxymethylbilane (HMB) by HemC. This HMB is then transformed into uroporphyrinogen III, a crucial intermediate in heme and B12 synthesis, by uroporphyrinogen III synthase. Our study identified significant upregulation (2.2–3.2-fold) of ALA and uroporphyrinogen III synthesis genes, specifically *hemA* (RM25_RS08900), *hemB* (RM25_RS08870), and *hemL2* (RM25_RS09040), under aerobic conditions during the later sampling point (III). However, the expression of genes involved in later steps of heme biosynthesis, such as RM25_RS09440 encoding putative uroporphyrinogen III synthase, *hemH* (RM25_RS08875), *hemY* (RM25_RS08880), and *hemE* (RM25_RS12355), did not show significant differential expression in response to atmospheric conditions (Table S1; Fig. 4). Furthermore, the expression of RM25_RS09440 was found to be affected by the growth stage and downregulated during the shift to the stationary phase under both atmospheric conditions.

### Transport of iron and α-ketoglutarate are increased under aerobic conditions

**Fig 5:**
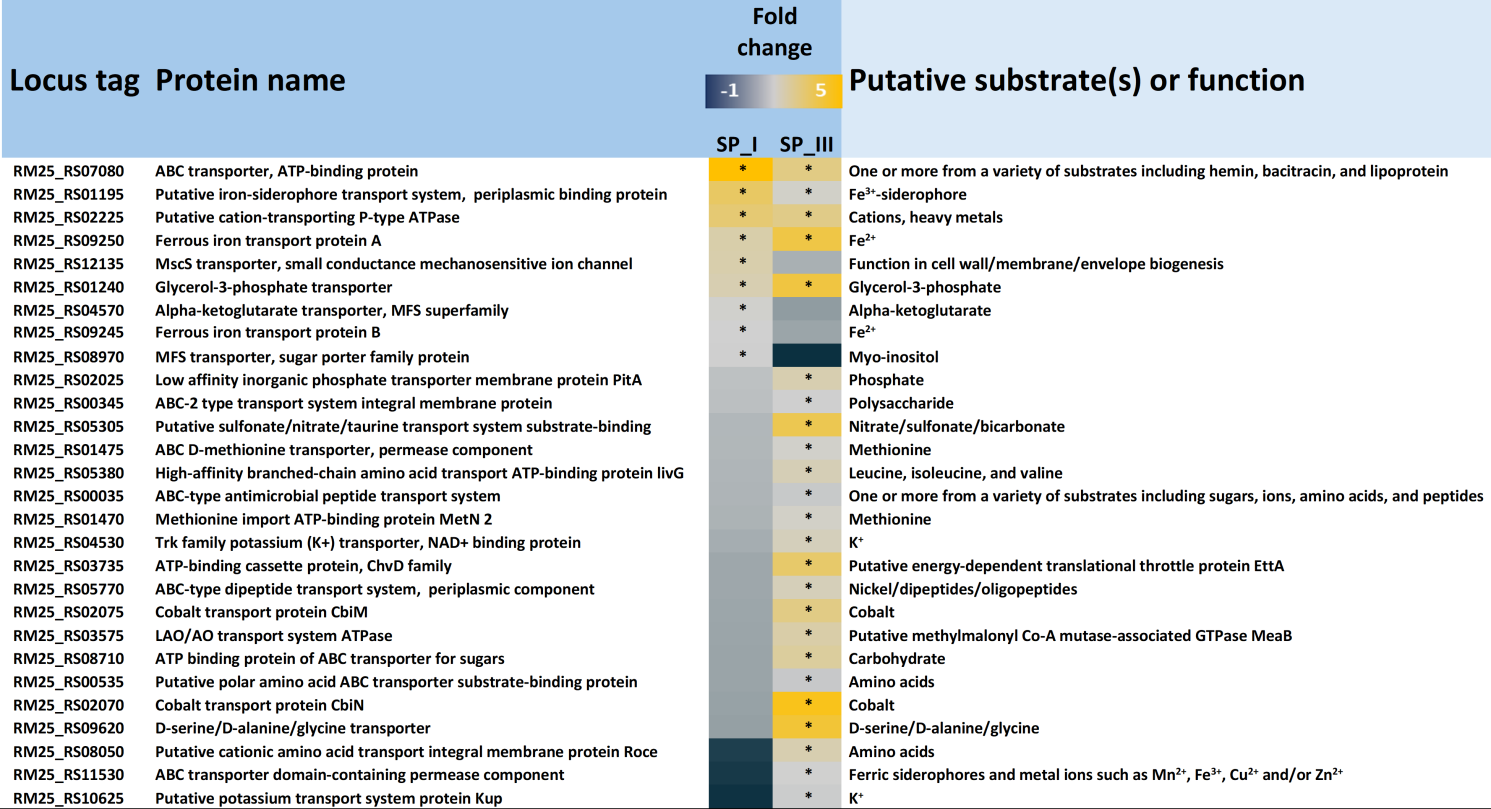
Genes associated with transport, import, or export that are upregulated (adjusted *P*-value ≤ 0.05) under aerobic conditions at Sampling Point I (SP_I) and/or Sampling Point III (SP_III) compared to anaerobic conditions. Genes exhibiting an upregulation fold change of ≥2.0 (log2FoldChange ≥ 1) are marked with an asterisk.

The results of the GO enrichment analysis revealed a significant impact on “transmembrane transport.” To delve deeper into this observation, genes from DSM 20271 were filtered based on annotations related to “transport,” “import,” or “export,” resulting in 208 genes potentially encoding transport functions. Within this pool of 208 genes, nine demonstrated more than a twofold upregulation under aerobic conditions at sampling point I, and 24 exhibited more than a twofold upregulation under aerobic conditions at sampling point III (Fig. 5). Conversely, among these 208 genes, 74 displayed more than a twofold upregulation under anaerobic conditions compared to aerobic conditions at sampling point I. Subsequently, at the later sampling point (III), 34 genes were observed to be upregulated under anaerobic conditions (Table S4).

Among the transport genes that exhibited upregulation during aerobic conditioning at sampling point I, three genes, *feoAB* and *yfmC* (RM25_RS09245, RM25_RS09250, and RM25_RS01195) are predicted to be involved in either iron uptake systems or siderophore-mediated iron uptake systems (Table S1; Fig. 4). Iron plays a vital role as an essential component of electron transport chains and respiratory enzymes, such as cytochromes, as well as in the functioning of catalases and peroxidases, which protect reactive oxygen species (ROS). Additionally, the gene *glpT* (RM25_RS01240), encoding the glycerol-3-phosphate transporter, was upregulated by 2.6- and 4.1-fold under aerobic conditions at sampling points I and III, respectively. Furthermore, the *kgtP_2* gene (RM25_RS04570), encoding the α-ketoglutarate permease, was upregulated under aerobic conditions. This permease facilitates the transport of α-ketoglutarate, a rate-determining intermediate in the TCA cycle, as well as a precursor of glutamate and thereby of porphyrin synthesis via the C5 pathway, which has emerged as a “master regulator metabolite,” as reviewed by Huergo et al. (64). Four other predicted transport genes that were upregulated under aerobic conditions at sampling point I are associated with the transportation of different molecules, including cations (RM25_RS02225), lipoproteins (RM25_RS07080), myo-inositol (RM25_RS08970), and ions (RM25_RS12135).

### Transfer of amino groups from aspartate/alanine to α-ketoglutarate is upregulated under aerobic conditions

While the majority of the amino acid metabolism-associated genes showed increased expression under anaerobic conditions (Table S1), the *alaT* gene (RM25_RS06515), coding for a putative alanine or aspartate transferase, exhibited over twice the expression under aerobic conditions compared to anaerobic conditions at both sampling points. This suggests that the enzyme activity, producing glutamate and either pyruvate or oxaloacetate, is more pronounced during aerobic growth. Among the anaerobically upregulated genes was *gadB*, which encodes glutamate decarboxylase, indicating a shift toward gamma-aminobutyric acid (GABA) production in the absence of oxygen (Fig. 4).

### The surfaceome of *PFR* is influenced by the atmosphere

As proteins exposed at the bacterial cell surface (surfaceome) play multiple roles in sensing, responding, and adapting to changing atmospheric conditions, we complemented the transcriptome data with surfaceomics analysis. We conducted an additional bioreactor cultivation analysis with *PFR* and harvested the cells at the mid-logarithmic growth phase under both the anaerobic (at 12-hour timepoint) and aerobic (at 13-hour timepoint) conditions. The washed cells were then subjected to trypsin-shaving and label-free quantitative (LFQ) identification, as detailed in Fig. 1.

#### Surface-proteins specific and common to aerobic and anaerobic conditions

**Fig 6:**
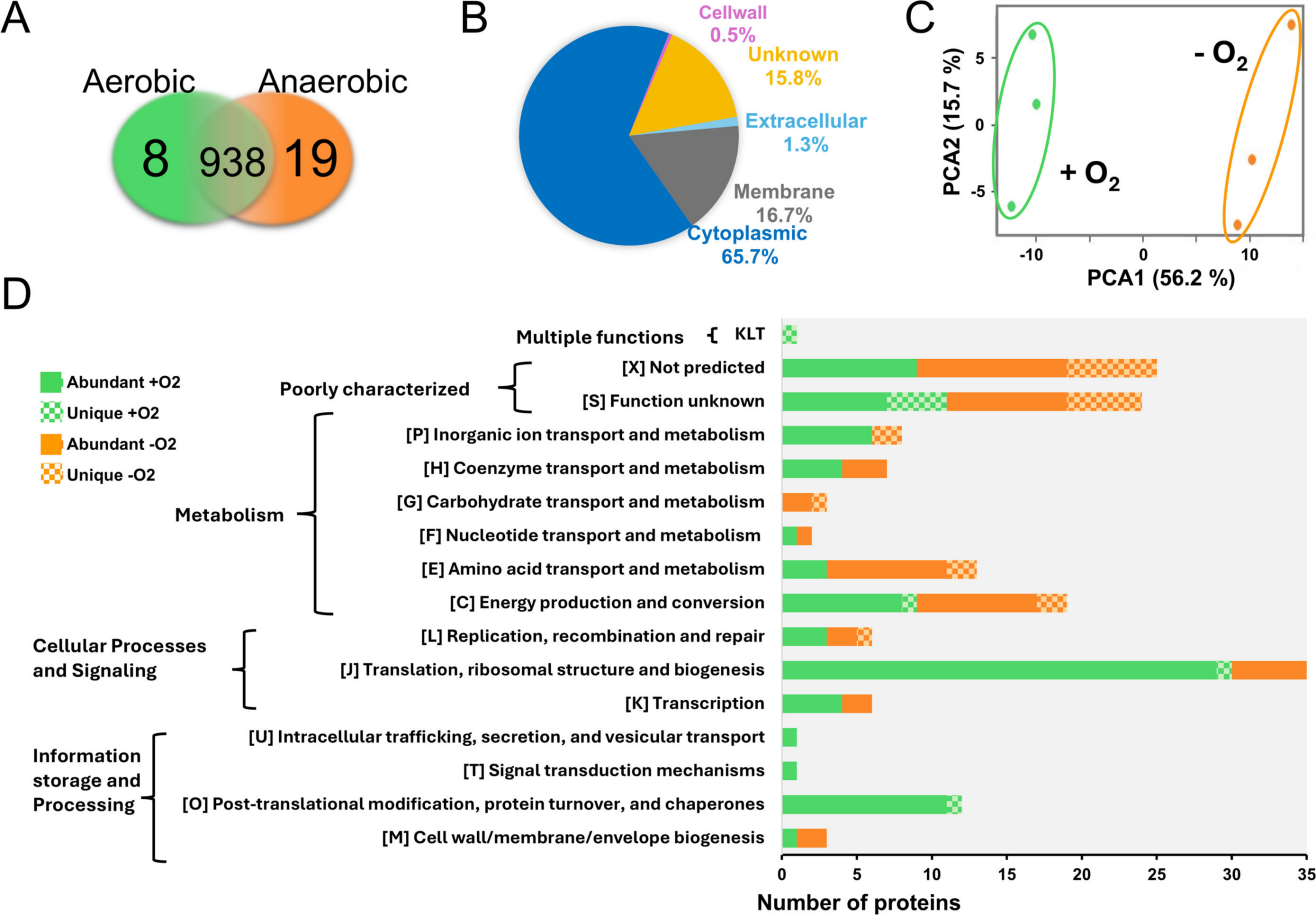
Proteomic analysis of *PFR* DSM 20271^T^ surfaceomes. (**A**) Venn diagram indicates uniquely and intersectively identified proteins in aerobic and anaerobic growth environments. (**B**) Subcellular localization of the identified surfaceomes (predicted by PSORTb v. 3.0.3). (**C**) The principal component analysis (PCA) plot of the LFQ intensities of the individual samples with three replicates. PCA identifies a correlation between the protein abundance profiles between aerobically (green) and anaerobically (orange) cultured bacterial cells. (**D**) Functional categorization of proteins that were either uniquely identified from aerobic (green) or anaerobic (orange) culture or differentially abundant between the two growth environments (predicted by eggNOG 5.0.0).

A total of 965 cell surface proteins were identified, along with their predicted subcellular locations and motifs, as listed in Table S5. Eight and nineteen proteins were exclusively identified in aerobic and anaerobic conditions, respectively (Fig. 6A; Table S5). These unique proteins, which exhibited low raw intensity values, were mostly detected in two out of three replicates with single-matching peptides, resulting in low sequence coverage. In mass spectrometry-based proteomics, raw intensity values reflect protein abundance (65–69). This suggests that while these proteins are present in low quantities in *PFR* cells, they are still more abundant than their counterparts, which remained below the detection limit under the opposite condition. These included a 30-kDa hypothetical protein specific to the aerobic surfaceome (RM25_RS12870) and a 12-kDa hypothetical protein specific to the anaerobic surfaceome (RM25_RS12605), which were detected with the highest raw intensity values among the uniquely identified proteins.

Other detected unique proteins included the following: (i) an argininosuccinate lyase (ASL) (RM25_RS06485) contributing to arginine production and integration of nitrogen metabolism with other metabolic pathways; (ii) a cobalt ABC transporter ATPase subunit (RM25_RS02060) involved in B12 synthesis; (iii) glutamine amidotransferase PdxT (RM25_RS07020) involved in nucleotide, amino acid, and coenzyme production as well as overall nitrogen metabolism; and (iv) an NarG subunit (RM25_RS10125) of the nitrate reductase (NR) complex involved in anaerobic respiration. All these were specific to the anaerobic surfaceome. The gene coding for NarG was also significantly upregulated (Table S1) under anaerobic conditions, implying the importance of NR in anaerobic respiration. Conversely, cobalt-associated transporter genes were upregulated during aerobic growth (Fig. 5), while a cobalt transporter ATPase (RM25_RS02060) was more abundant under anaerobic conditions at the surfaceomic level. Nineteen unique IDs originated from the cytoplasm, and six were predicted to harbor signals/motifs anchoring the protein to the cell wall or membrane (Table S5). Among the identified proteins, 938 were present in both the aerobic and anaerobic surfaceomes (Fig. 6A). Of these, 85 proteins were predicted to harbor a signal peptide (Sec/SPI, Sec/SPII, or Tat/SPI type), and 79 proteins with 2–14 TMDs, directing the protein either out of the cells or into the cell membrane, respectively. Cytoplasmic proteins comprised the largest group (n = 634) (Fig 6B), which aligns well with other studies showing that cytoplasmic proteins on the cell surface dominate over those anchored to the cell wall or membrane through motifs or domains (70–78). Several factors could explain the presence of predicted cytoplasmic proteins within the surfaceome: (i) destabilization of biological membranes caused by the trypsin treatment (75, 78, 79); (ii) controlled cell lysis (via autolysins, hydrolases, or phages) and normal cell turnover (80, 81); (iii) cellular remodeling and release of extracellular vesicles (EVs) (82, 83); and (iv) low environmental pH promoting adherence of cytoplasmic proteins to the cell wall structures (84).

#### Peptidoglycan-degrading enzymes are most abundant on PFR cell surfaces

High-abundance surface proteins can play a crucial role during physiological growth and interactions with the environment, so the proteins are listed according to their raw intensity values in Table S5 (from lines 28 to 957). Most of the proteins identified with the highest intensity values here have been previously detected in other surfaceome/secretome studies on *PFR* (4, 85). The most abundant proteins included an LytG, an NlpC/P60 family peptidase, and a peptidoglycan hydrolase—RpfA, which were predicted to be secreted via the classical signal peptide-dependent pathway (Sec/SPI). Other abundant proteins included three cytoplasmic proteins, the chaperones GroL1_1, GroL1_2, and the elongation factor—EfTU, proposed to reach the cell surface through a non-classical, yet unidentified mechanism. Both RpfA and NlpC/P60 peptidase have been shown to contribute to peptidoglycan hydrolysis (86, 87). Thus, these enzymes could be involved in the controlled release of the chaperons and the elongation factor to the cell surface of *PFR*. These proteins belong to a group of typical cytosolic moonlighting proteins with additional functions in many organisms (88). For example, GroL1 proteins could have contributed to adherence, sensing/signaling, antiadipogenic, and even iron-binding activities, at the cell surface beyond their classical chaperone function within the cell, as demonstrated for *PFR* and other bacteria (89–92).

#### Condition-dependent protein abundance changes

Principal component analysis (PCA) of the LFQ intensities of each detected cell surface protein (Fig. 6A) explains over 70% of the total variation and indicates clear clustering of the aerobic and anaerobic surfaceomes. The biological replicates within each condition demonstrate a strong negative correlation in aerobic conditions and strong positive correlation in anaerobic conditions, indicating that *PFR* responds to oxygen level changes by altering the abundance of specific cell surface proteins, potentially through gene expression changes or modifications in protein secretion and stability.

A two-sample *t*-test (FDR 0.05) revealed that the LFQ intensities of 139 proteins significantly differed between the two growth conditions (Table S6). Among these, 88 proteins were more abundant on the aerobic surfaceome, while 51 proteins were more abundant in anaerobic conditions. The most abundant proteins included RpfA peptidoglycan hydrolase and the GroL1 chaperones, which were significantly more abundant in the aerobic surfaceome, while NlpC/P60 peptidase and the elongation factor: EfTu were more abundant in anaerobic conditions (Tables S5 and S6). The majority of the condition-dependent changes involved cytoplasmic proteins, many of which have reported secondary function outside of the cells, beyond their classical intracellular roles (90). The largest proportion of differentially abundant surface proteins (*n* = 139) were involved in translation, ribosomal structure, and biogenesis (Fig. 6D). This finding aligns with those of previous studies that emphasize the prominent role of this functional category at the bacterial cell surface (79, 93–96). This included r-proteins that were more abundant in aerobic surfaceome than the anaerobic one. Since their gene expression did not change between the tested conditions, the abundance difference at the cell surface is likely not due to differential gene expression. A similar observation has been made in *Lacticaseibacillus rhamnosus* GG, where an r-protein L2 (RPL2) showed bile-dependent abundance changes (95).

Possible explanations for condition-specific increase in the cytoplasmic protein abundance include the RpfA-mediated protein release and physicochemical changes at the cell surface, which promote the binding of r-proteins. These proteins, which showed oxygen-stimulated abundance increase (Tables S5 and S6), may also help bacteria adjust their cell surface hydrophobicity and charge in response to environmental changes (97, 98). For example, r-proteins can enhance biofilm integrity in Gram-positive *Staphylococcus aureus* due to their strong positive charge in acidic conditions (72). Given that r-proteins are positively charged at physiological pH (97), we suggest that external oxygen may alter the net charge or reduce the cell surface hydrophobicity (99–101) in *PFR* cells, enhancing the binding of cytoplasmic proteins, including r-proteins, under aerobic conditions. Studies have also demonstrated that drop in environmental pH promotes the binding of several cytoplasmic proteins to cell surface structures (84, 102).

Interestingly, studies on the EV proteins of different *S. aureus* strains indicate that 36%–60% of the proteins are cytoplasmic (103). For *PFR* EVs, this figure is reported to exceed 70% (83), suggesting that the bacteria use both selective and passive sorting mechanisms, with high-abundance proteins more likely to be loaded into EVs (83, 103, 104). Most of the r-proteins showing changes in abundance have been identified in EVs produced by *PFR* (5, 82, 83) and other bacteria (105–108), implying that the tryptic-shaving conditions used in this study were able to capture these structures during their exit from the cells. Rodovalho et al. (5) proposed that *PFR* might increase EV production in response to environmental stress, supported by studies on *L. reuteri* DSM 17938, where oxygen stress influenced the number and protein concentration of EVs (107). Thus, we suggest that some cytoplasmic proteins are exported via a controlled process involving both EVs and cell lysis, as r-protein abundance changes were condition-dependent, while gene expression remained constant under the tested conditions.

Two of the most abundant cytoplasmic moonlightning proteins, the GroL chaperones, showed an oxygen-stimulated increase in *PFR* (Tables S5 and S6). These chaperones have been reported to bind iron in other bacteria (109). This moonlighting function could benefit *PFR* in aerobic conditions, as iron is a key component of cytochromes and other proteins involved in the electron transport chain, crucial for energy production during aerobic respiration. Related proteins showing oxygen-dependent increases included potential aerobic respiration proteins NuoC1 (RM25_RS02345) and Nqo4 (RM25_RS02350), FrdA2 (RM25_RS06660) and FrdB2 (RM25_RS06655), and CydA (RM25_RS00880) of NADH hydrogenase, succinate dehydrogenase (SDH), and cytochrome bd complexes, respectively, in addition to AtpF (RM25_RS05585) of ATP synthase (Table S5). In contrast, the DmsA (RM25_RS09695) and DmsB (RM25_RS09690) subunits of dimethyl sulfoxide (DMSO) reductase and the uniquely detected NarG of the NR complex were more abundant on the anaerobic surfaceomes (Tables S5 and S6; Fig. 4). Genes encoding SDH were upregulated under aerobic, while *narG* was upregulated under anaerobic conditions (Table S1). These proteins are either classically anchored to the cell wall/membrane via specific motifs/domains (CydA, AtpF, and DmsA) or are part of a membrane-spanning protein complex (NuoC1, Nqo4, FrdA2, FrdB2, DmsB, and NarG), justifying comparisons of gene expression and protein abundance levels. Our findings suggest that in the presence of oxygen, aerobic respiration takes precedence for electron transfer-linked phosphorylation, whereas in its absence, alternative acceptors like nitrate and DMSO may be utilized.

#### Increasing availability of key compounds and metabolites can enhance aerobic growth of PFR

**Fig 7:**
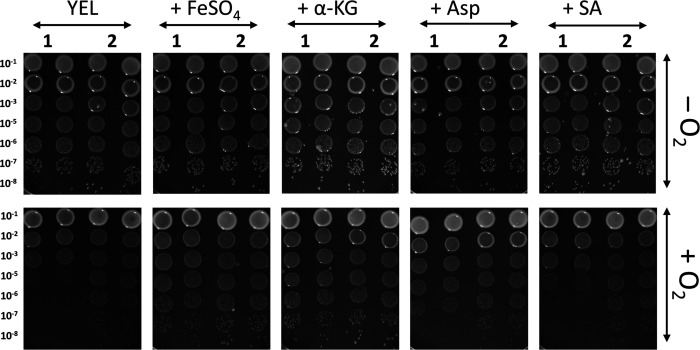
Colony formation of DSM 4902 (1) and DSM 20271 (2) under anaerobic (-O2) and aerobic (+O2) conditions on YEL agar plates with and without supplementation of FeSO_4_ (1 mM), aspartic acid (10 mM), α-ketoglutaric acid (1 mM), or succinic acid (1 mM) after 4 (anaerobic) or 6 (aerobic) days of incubation at 30°C. The images are representative of experiments repeated at least three times for each condition. YEL, yeast extract–lactate medium; a-KG, a-ketoglutarate; Asp, aspartic acid; SA, succinic acid.

Omics data indicate that pathways involving iron and α-ketoglutarate transport and metabolism, as well as succinate metabolism, are more active in aerobic conditions. These data also suggest that alanine/aspartate transferase activity is increased when *PFR* is exposed to oxygen. Aspartate aminotransferase catalyzes the transamination of aspartate and α-ketoglutarate, forming glutamate and oxaloacetate, both of which are intermediate metabolites in the TCA cycle, alongside α-ketoglutarate and succinate. Thus, it could be that increased availability of iron, aspartate, α-ketoglutarate, or succinate influences the aerobic growth of *PFR*. To test this, the colony formation of DSM 20271 together with another *PFR* type strain (DSM 4902) on YEL agar with and without FeSO_4_, aspartate, α-ketoglutarate, or succinate both under aerobic and anaerobic conditions was compared. Earlier studies reported an inability of *PFR* to form colonies under aerobic conditions (110, 111), whereas a recent study demonstrated that DSM 20271 thrives in the presence of oxygen (13), implying strain-dependent differences in cell response to oxygen. Our results revealed that under anaerobic conditions, 4 days after incubation at 30°C, colonies were formed equally by both strains. However, after 6 days of incubation under aerobic conditions, no visible separate colonies of DSM 4902 formed without supplementation, whereas miniscule colonies of DSM 20271 were observed (Fig. 7). Notably, the addition of FeSO_4_ and α-ketoglutarate significantly improved the colony-forming ability of DSM 4902, while aspartate and succinate had a lesser effect (Fig. 7). Moreover, DSM 20271 formed small colonies under aerobic conditions even without supplementation. The addition of FeSO_4_ and α-ketoglutaric acid increased the size of DSM 20271 colonies under aerobic conditions (Fig. 7). α-ketoglutarate and succinate are important intermediates in the TCA cycle, while aspartic acid can be metabolized in various ways, including conversion to another TCA intermediate, oxaloacetate. Thus, it is tempting to suggest that the addition of these metabolites in YEL enhances the flow of carbon and energy through the TCA cycle, thereby promoting growth and colony-forming ability under aerobic conditions. On the other hand, iron is crucial for various cellular processes, including electron transport in the respiratory chain. Thus, iron sulfate can help improve the efficiency of energy production by supporting the function of iron-containing enzymes in the bacterium’s electron transport chain.

#### FeSO_4_ controls the production of heme in the presence of oxygen

FeSO₄ supplementation was found to enhance the colony formation ability under aerobic conditions, potentially through increased heme production and the activity of heme-dependent enzymes (e.g., cytochromes, catalase, and peroxides). To further study the effect of oxygen on heme production and its connection to B12 production, DSM 20271 was cultured in triplicate for 96 hours under microaerobic conditions, with and without 1 mM FeSO₄. Control cultures were propagated anaerobically.

**Fig 8:**
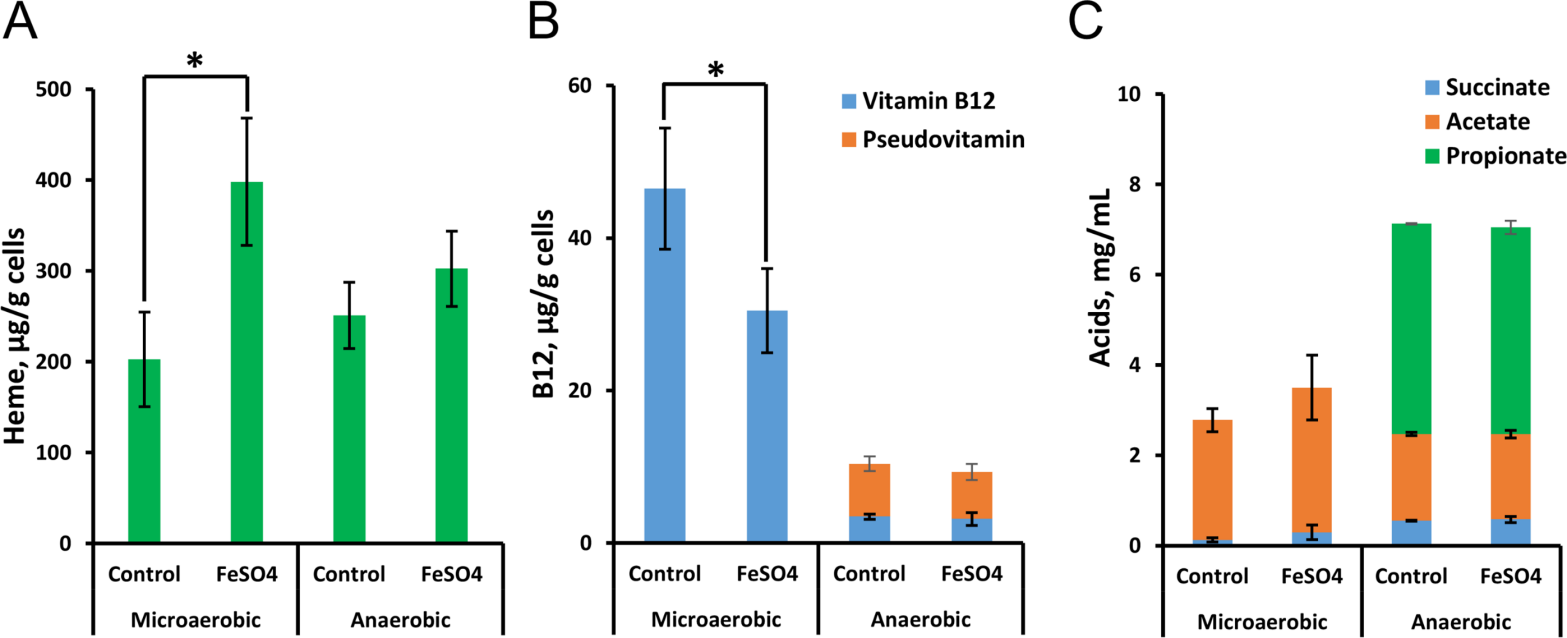
Effect of FeSO_4_ (1 mM) supplementation on the biosynthesis of heme (**A**), vitamin B12 (**B**), and accumulation of organic acids (**C**) when grown under microaerobic and anaerobic conditions in yeast extract–lactate medium supplemented with CoCl_2_ (5 mg/L). The values are means and standard deviations of three biological replicates. A significant difference between the treatments (*P* < 0.05) is indicated by an asterisk.

Results revealed no impact on final cell densities with FeSO₄ supplementation (data not shown). However, under microaerobic conditions, the addition of FeSO₄ elevated heme production and reduced B12 production (Fig. 8). This suggests a shift in tetrapyrrole synthesis toward heme in the presence of excess oxygen and iron, at the expense of B12 production. Superior B12 production under microaerobic conditions aligns with a previous report by Dank et al. (14) as well as our bioreactor results (Fig. S1B and F). Notably, the cobamide detected under microaerobic conditions is the B12 form, while both pseudo-B12 and B12 forms are produced under anaerobic conditions (Fig. 8). Acetate and succinate were present in all samples, while propionic acid was exclusive to anaerobic conditions, indicating potential differences in propionic acid metabolism under microaerobic conditions (13).

### Conclusions

We investigated the metabolism and growth characteristics of *PFR* DSM 20271 under aerobic and anaerobic conditions. Our results showed enhanced biomass formation under aerobic conditions. Additionally, lactate consumption was faster under anaerobic conditions, while propionate and acetate levels increased over time under anaerobic conditions but remained low under aerobic conditions. Cobamide synthesis was notably impacted by oxygen, with minimal B12 or pseudo-B12 production under aerobic conditions compared to anaerobic or microaerobic conditions. Genes related to iron and α-ketoglutarate transport and metabolism, succinate metabolism, and alanine/aspartate transferase activity were found upregulated under aerobic conditions, indicating energy production through respiration and classifying *PFR* as a facultative anaerobe. Surfaceome analyses revealed an oxygen-dependent increase in peptidoglycan-hydrolyzing enzymes (RpfA and LytG) along with several r-proteins and other cytoplasmic proteins. This suggests that *PFR* may utilize controlled cell lysis or vesiculation to release cytoplasmic moonlighting proteins, influenced by changes in cell surface structures or gene expression triggered by oxygen. Moreover, our study demonstrated that the growth of DSM 20271 under aerobic conditions could be enhanced by supplementation with FeSO_4_, aspartate, α-ketoglutarate, or succinate. Interestingly, FeSO_4_ supplementation also increased heme production at the expense of B12 production in the presence of oxygen, suggesting a negative effect of externally available iron on B12 synthesis in *PFR*.

Future research should include additional strains and experimental setups as the current bioreactor conditions limit the generalizability of the findings. The impact of oxygen and iron availability on metabolic pathways, such as heme and B12 production, should be studied in more detail to understand the effects of different environmental factors. Furthermore, integrating other omics approaches, such as total proteomics and metabolomics, could provide a more comprehensive understanding of the bacterium’s physiological responses.

In summary, our findings underscore the significant role of oxygen in the metabolism and growth of *P. freudenreichii* DSM 20271. These insights have potential practical implications for improving cell mass in starter and probiotic culture production and increasing heme production by *PFR*, with possible applications in the development of nutritionally rich food products that serve as alternative protein sources.

## Data Availability

The transcriptomics and proteomics data sets generated during and/or analyzed during the current study are available in the SRA database under the accession PRJNA872337 and PRIDE repository with data set identifier PXD051703. The other data sets generated during and/or analyzed during the current study and not included in the article are available from the corresponding author on reasonable request.

## SUPPLEMENTAL MATERIAL

Fig. S1 msystems.00615-24-s0001.docx Aerobic vs. microaerobic bioreactor growth experiments.

Additional experimental details msystems.00615-24-s0002.docx Validation of RNAseq data with ddPCR.

Captions msystems.00615-24-s0003.docx Captions for supplemental tables.

Table S1 msystems.00615-24-s0004.xlsx Results of RNA sequencing of the strain DSM 20271 grown in bioreactors under anaerobic and aerobic atmospheres.

Table S2 msystems.00615-24-s0005.xlsx DEGs across sampling points I and III.

Table S3 msystems.00615-24-s0006.xlsx Results of GO category analysis.

Table S4 msystems.00615-24-s0007.xlsx Genes encoding transport proteins upregulated under anaerobic conditions.

Table S5 msystems.00615-24-s0008.xlsx List of all identified proteins.

Table S6 msystems.00615-24-s0009.xlsx List of proteins showing statistically significant protein abundance change between the aerobic and anaerobic surfaceomes.

ASM does not own the copyrights to Supplemental Material that may be linked to, or accessed through, an article. The authors have granted ASM a non-exclusive, world-wide license to publish the Supplemental Material files. Please contact the corresponding author directly for reuse.
