# Supplementary material for: Aerobic adaptation and metabolic dynamics of Propionibacterium freudenreichii DSM 20271: insights from comparative transcriptomics and surfaceome analysis
Source: mSystems. 2024 Sep 30;9(10):e00615-24. doi: 10.1128/msystems.00615-24 (PMC11494915; doi:10.1128/msystems.00615-24)
Supplement: Fig. S1 — Aerobic vs. microaerobic bioreactor growth experiments. [file msystems.00615-24-s0001.docx]

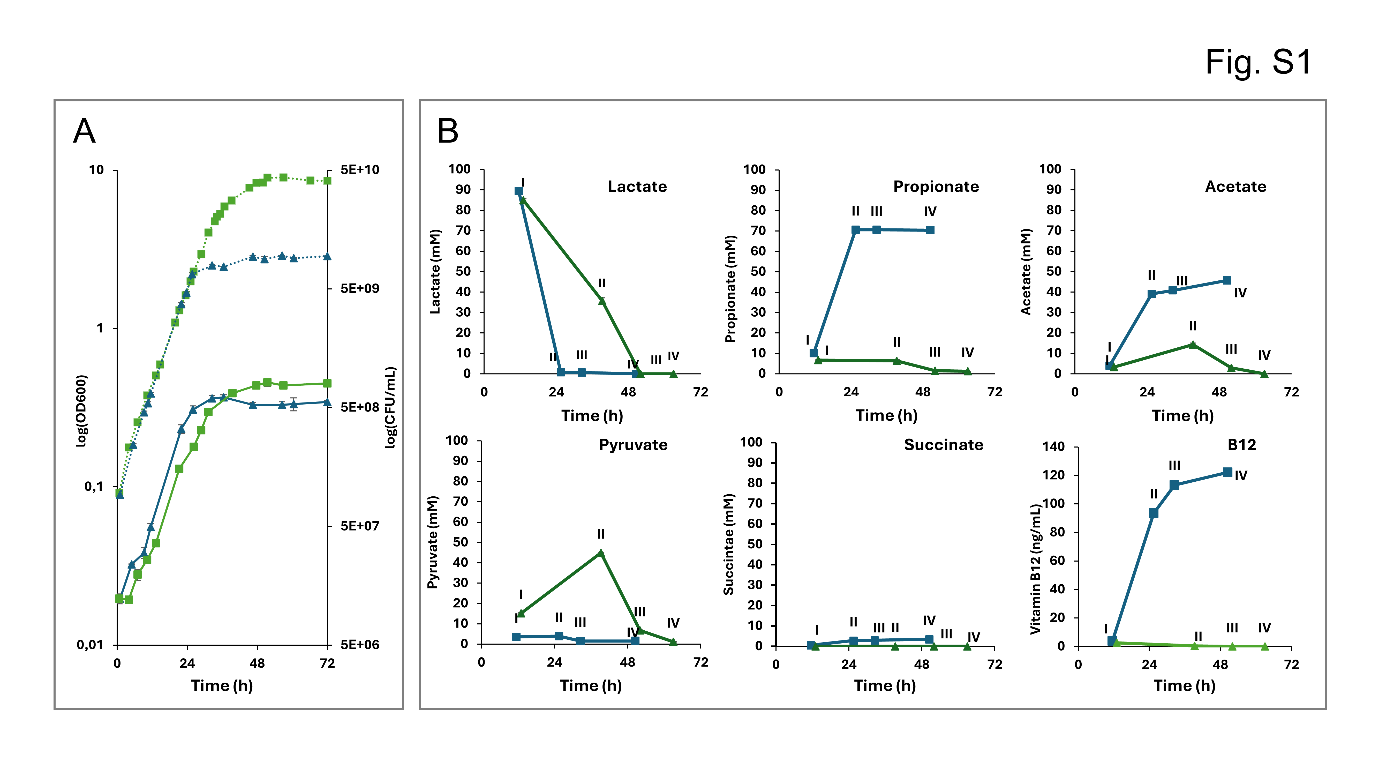


**Supplementary figure**

**Figure_S1.** (A) Cell densities (OD_600_ values - solid line) and cell viability (CFU/mL - dotted line) in bioreactors conducted experimented under aerobic (green) and microaerobic (blue) growth conditions. (B) Lactate utilization (A), excreted propionate (B), acetate (C), pyruvate (D), succinate (E) and intracellular vitamin B12 of *P. freudenreichii* DSM 20271 during cultivation under aerobic (green) and microaerobic (blue) fermentations performed in bioreactors. n = 3. In aerobic fermentation, samples were collected at 13 h (I), 40 h (II), 52 h (III), and 66 h (IV). For microaerobic fermentation samples were obtained at 12 h (I), 26 h (II), 22 h (III), and 51 h (IV).
