# Supplementary material for: Aerobic adaptation and metabolic dynamics of Propionibacterium freudenreichii DSM 20271: insights from comparative transcriptomics and surfaceome analysis
Source: mSystems. 2024 Sep 30;9(10):e00615-24. doi: 10.1128/msystems.00615-24 (PMC11494915; doi:10.1128/msystems.00615-24)
Supplement: Captions — for supplemental tables. [file msystems.00615-24-s0003.docx]

**Supplementary Table legends and References**

**Table S1.** **Results of RNA sequencing of the strain** P. freudenreichii **DSM 20271 grown in bioreactors under anaerobic (nitrogen) and aerobic (oxygen) atmospheres at sampling points I and III. Columns A-I specify the order of annotated genes, locus tags of the current NCBI annotations, the previous version of the NCBI locus tags, product names, locations in the genome and strand orientation. Column J-AM: results of DEseq2 analyses with models comparing the effect of atmosphere (anaerobic vs. aerobic) at both sampling points (columns J-O); the effect of atmosphere (aerobic vs. anaerobic) at sampling point I (columns P-U); the effect of atmosphere (aerobic vs. anaerobic) at sampling point III (columns V-AA); the effect of growth phase under anaerobic atmosphere (columns AB-AG) and the effect of growth phase under aerobic atmosphere (AH-AM). Columns AN-BA list results from previously reported bioinformatics analyses (1), including annotations with PROKKA, core genes identified through comparative analyses against other** PFR **strains with ROARY, IslandViewer, Prophinder, Phaster, REBASE and CRISPRFinder.**

**Table S2. Differentially expressed genes (DEGs) across sampling points I and III. Out of 662 upregulated DEGs identified under aerobic conditions at sampling point I (SPI), 300 remained upregulated at sampling point III (SPIII) (Columns A-B), while 19 were downregulated at sampling point III (Columns C-D). Among the 713 downregulated DEGs at SPI, 277 were consistently downregulated at SPIII (Columns E-F), and 11 were found to be upregulated (Columns G-H). Columns I-P specify DEGs that are uniquely upregulated or downregulated at either SPII or SPIII. Genes were considered significantly differentially expressed if their adjusted p-value (padj) was ≤ 0.05 and the fold changes were ≥ 2.0 (log2Fold ≥ 1). The lists include both the the current NCBI locus tags and the previous version of the NCBI locus tags.**

**Table S3. Results of GO Category Analysis. Genes were considered significantly differentially expressed if their adjusted p-value (padj) was ≤ 0.05. Predicted Gene Ontology (GO) terms for these genes were obtained using PANNZER2 (2) with default parameters. The lists of differentially expressed genes from the various comparisons were analyzed for GO term enrichment across the Biological Process, Molecular Function, and Cellular Component ontologies using the R package clusterProfiler (v. 4.0) (3, 4). The reference set for this analysis included all genes annotated with GO terms in the genome. GO categories were compared at sampling points I and III, and those showing statistically significant differences (p < 0.05) under different conditions were listed (Column A). The table details the genes within each GO category, including their upregulated or downregulated status. Column B: Numerical GO term; Column C: p-value; Column D: Category name; Column E: Functional category; Column F: Total number of genes in the category. Columns G-I: Number and percentage of upregulated genes, along with their corresponding list; Columns J-L: Number and percentage of downregulated genes and their corresponding list.**

**Table S4.** **List of 74 genes encoding transport proteins detected with more than two-fold higher expression under anaerobic conditions.**

**Table S5.** List of all identified proteins with the detected raw intensity values for each. Reverse hits, potential contaminants, and proteins only identified by site were filtered out. Proteins were filtered to contain minimally 2/3 valid values in at least one of the growth environments. The number of transmembrane spanning domains (TMD) was predicted using TMHMM 2.0, subcellular localization of proteins was done with prediction tool PSORTb 3.0.3, and the presence of possible classical and non-classical signal peptide sequences was analyzed with LipoP 1.0, SignalP 5.0, and SecretomeP 2.0. The COG categorization was accomplished with EggNOG 5.0.0. Proteins in bold and green font were specific to the aerobic surfaceome. Proteins in bold and red were specific to the anaerobic surfaceome.

**Table_S6.** List of proteins showing statistically significant protein abundance change between the aerobic and anaerobic surfaceomes. For this purpose, the LFQ intensities after log2 conversion and missing value imputation were subjected to two-sample t-test with a permutation-based false discovery rate of 0.05 to confirm the statistical significance. The number of transmembrane spanning domains (TMD) were predicted using TMHMM 2.0, subcellular localization of proteins were done with prediction tool PSORTb 3.0.3 PSORTb v3.0.2, and the presence of possible classical and non-classical signal peptide sequences were analysed with LipoP 1.0, SignalP 5.0, and SecretomeP 2.0. The COG categorization was accomplished with EggNOG 5.0.0.

**References**

1. Deptula P, Laine PK, Roberts RJ, Smolander O-P, Vihinen H, Piironen V, Paulin L, Jokitalo E, Savijoki K, Auvinen P. 2017. De novo assembly of genomes from long sequence reads reveals uncharted territories of Propionibacterium freudenreichii. BMC Genom 18:1-24.
2. Törönen P, Holm L. 2022. PANNZER-A practical tool for protein function prediction. Protein Sci 31:118-128.
3. Wu T, Hu E, Xu S, Chen M, Guo P, Dai Z, Feng T, Zhou L, Tang W, Zhan L, Fu X, Liu S, Bo X, Yu G. 2021. clusterProfiler 4.0: A universal enrichment tool for interpreting omics data. The Innovation 2:100141.
4. Yu G, Wang LG, Han Y, He QY. 2012. clusterProfiler: an R package for comparing biological themes among gene clusters. Omics 16:284-7.
5. Untergasser A, Nijveen H, Rao X, Bisseling T, Geurts R, Leunissen JA. 2007. Primer3Plus, an enhanced web interface to Primer3. Nucleic Acids Res 35:W71-4.
